# Determination of Coenzyme Q10 Content in Food By-Products and Waste by High-Performance Liquid Chromatography Coupled with Diode Array Detection

**DOI:** 10.3390/foods12122296

**Published:** 2023-06-07

**Authors:** Cristina Anamaria Semeniuc, Floricuța Ranga, Andersina Simina Podar, Simona Raluca Ionescu, Maria-Ioana Socaciu, Melinda Fogarasi, Anca Corina Fărcaș, Dan Cristian Vodnar, Sonia Ancuța Socaci

**Affiliations:** 1 Faculty of Food Science and Technology, University of Agricultural Sciences and Veterinary Medicine of Cluj-Napoca, 3-5 Mănăştur St., 400372 Cluj-Napoca, Romania; cristina.semeniuc@usamvcluj.ro (C.A.S.); floricutza_ro@yahoo.com (F.R.); rallucab@yahoo.com (S.R.I.); maria-ioana.socaciu@usamvcluj.ro (M.-I.S.); melinda.fogarasi@usamvcluj.ro (M.F.); anca.farcas@usamvcluj.ro (A.C.F.); dan.vodnar@usamvcluj.ro (D.C.V.); 2 Centre for Technology Transfer-BioTech, 64 Calea Florești, 400509 Cluj-Napoca, Romania

**Keywords:** coenzyme Q10, food by-products, food waste, ultrasonic extraction, 2-propanol extracts, high-performance liquid chromatography, diode-array detection

## Abstract

Coenzyme Q10 (CoQ10) is a vitamin-like compound found naturally in plant- and animal-derived materials. This study aimed to determine the level of CoQ10 in some food by-products (oil press cakes) and waste (fish meat and chicken hearts) to recover this compound for further use as a dietary supplement. The analytical method involved ultrasonic extraction using 2-propanol, followed by high-performance liquid chromatography with diode array detection (HPLC-DAD). The HPLC-DAD method was validated in terms of linearity and measuring range, limits of detection (LOD) and quantification (LOQ), trueness, and precision. As a result, the calibration curve of CoQ10 was linear over the concentration range of 1–200 µg/mL, with an LOD of 22 µg/mL and an LOQ of 0.65 µg/mL. The CoQ10 content varied from not detected in the hempseed press cake and the fish meat to 84.80 µg/g in the pumpkin press cake and 383.25 µg/g in the lyophilized chicken hearts; very good recovery rates and relative standard deviations (RSDs) were obtained for the pumpkin press cake (100.9–116.0% with RSDs between 0.05–0.2%) and the chicken hearts (99.3–106.9% CH with RSDs between 0.5–0.7%), showing the analytical method’s trueness and precision and thus its accuracy. In conclusion, a simple and reliable method for determining CoQ10 levels has been developed here.

## 1. Introduction

Coenzyme Q10 (CoQ10) is a lipid-soluble molecule found in the mitochondria of each cell of eukaryotic and prokaryotic organisms [1,2]. It is involved in mitochondrial processes, such as respiration, cellular ATP (adenosine triphosphate) synthesis, maintaining heart muscle strength, neutralizing free radicals in the fight against aging, and stimulating the immune system [3]. With a chemical structure of 2,3-dimethoxy-5-methyl-6-decaprenyl-1,4-benzoquinone, CoQ10 is also known as ubiquinone-10 due to the “ubiquitous” occurrence of this compound in cells [4,5,6]. CoQ10 is also present in food products but is denatured by thermal processing because it is thermosensitive [7,8].

A previous paper [9], a literature review, highlighted vegetable oils, fish oil, organs, and meat as the richest sources of CoQ10. Based on this information, press cakes could be considered potential sources of this compound, given that they still contain oils, and cold-press oil extraction creates high amounts of these by-products [10]. In addition, the meat and fish industries generate large quantities of solid waste [11,12] since their primary products have a short shelf-life when stored in refrigerated conditions. Therefore, it would be appropriate to investigate whether fish meat and chicken hearts from supermarket shelves still contain CoQ10 after expiration when they are considered waste.

Several methods are available in the literature for CoQ10 determination in food matrices by high-performance liquid chromatography with diode array detection (HPLC-DAD) [8,13,14,15,16,17,18], which first consists of analyte extraction with different solvents, followed by the instrumental analysis of the obtained extracts [9]. These include direct extraction with an ethanol/hexane mixture [13,14,15,18], supercritical carbon dioxide extraction [16], accelerated solvent extraction [17], and extraction by saponification [8]. Choosing the appropriate analytical method for a given problem must consider the analyst’s needs which must be weighed against the advantages and disadvantages of the available techniques [19]. In addition, revalidation or verification is necessary whenever a method is changed or applied to a new circumstance (such as a different sample matrix), depending on the change’s degree and the unique circumstance’s nature [20]. Therefore, typical characteristics such as accuracy, precision, specificity, detection limit, quantification limit, linearity, and range should be considered when validating an analytical method [21]. The extraction methods mentioned above are suitable for isolating CoQ10 from food matrices. However, some of these employ significant amounts of hazardous reagents, negatively impacting the environment and human health. As a result, they generate large quantities of toxic chemical waste. Therefore, it is preferable to obtain extracts by avoiding or reducing toxic solvents to minimize the side effects of analytical methods [17].

Isopropanol (2-propanol or propan-2-ol), a polar solvent, can also be used as a CoQ10 extractant from food matrices [22,23]. It is at the top of the list of green chemicals and is considered environmentally safe in industrial solvent selection guidelines [24]. Moreover, CoQ10 is more soluble in 2-propanol than in ethanol, while triglycerides, possible interference compounds of the food matrix, are not very soluble in this solvent [15]. To increase the yield of CoQ10, before extraction, sample pretreatment with ultrasound can also be applied [25]. According to Directive 2009/32/EC of the European Parliament and the Council [26], 2-propanol is already approved as an extraction solvent for processing raw materials, foodstuffs, food components, or ingredients, with a maximum residue limit of 10 mg/kg food in the extracted foodstuff or food ingredient. Assuming that it is used in soft drinks at a concentration of 600 mg/l, in 2005, the European Food Safety Authority’s Scientific Panel on food additives, flavorings, processing aids, and materials in contact with food estimated a mean potential consumption of 1.3 mg/kg bw/day, which is below the acceptable daily intake [27].

This study aims to assess the feasibility of using some food by-products (oil press cakes) and waste (fish meat and chicken hearts), presumed to be rich in CoQ10, as natural sources of this bioactive compound. It, therefore, proposes an analytical procedure for recovering and quantifying CoQ10 from such matrices through ultrasonic extraction with 2-propanol, preceded by HPLC coupled to DA detection. For method validation, the experimental design also includes linearity and spike-and-recovery experiments. No studies have so far investigated the CoQ10 content in oil press cakes using an HPLC-DAD technique. Altogether, the findings of this study provide valuable information for companies in the extractive industries interested in the commercial-scale extraction of CoQ10 from vegetable and animal materials.

## 2. Materials and Methods

### 2.1. Sample Collection

The chicken hearts were purchased from S.C. Puiul Regal S.R.L. (Gilău, Romania) in a quantity of 2.1 kg on 30 May 2022 and kept in a refrigerator (at 4 °C) until the expiration date written on the packaging, 4 June 2022, when they became waste. On the day following expiry (5 June 2022), they were minced with a meat grinder (N12; Lancom Distribution S.R.L., Bucharest, Romania), homogenized using a silicone spatula, divided into 50 and 100 g portions, and then stored in sealed polyethylene bags. Six 100 g shares were lyophilized in a laboratory freeze-dryer (LyoQuest-55; Azbil Telstar Technologies S.L., Barcelona, Spain) under the following operating conditions [28], and the rest of them were kept at −18 °C until extraction:

- Freezing conditions: freezing temperature −80 °C and freezing time 24 h;
- Sublimation conditions: vacuum pressure 0.01 mbar, sublimation temperature −55 °C, and sublimation time ~3 days (until a constant weight (±0.005 g)). The lyophilization yield was 22.1%.

The fish, chilled whole rainbow trout (*Oncorhynchus mykiss*), were purchased from Bistromar La Timona S.R.L. (Bucharest, Romania) in a quantity of 1.573 kg on 3 June 2022 and kept in a refrigerator (at 4 °C) until the expiration date written on the packaging, 4 June 2022, when they became waste. The next day (5 June 20220), the fish individuals were cut into pieces using a stainless-steel knife, then minced with a meat grinder (N12; Lancom Distribution S.R.L., Bucharest, Romania), homogenized using a silicone spatula, divided into 50 and 100 g portions, and stored in sealed polyethylene bags. Six parts of 100 g were lyophilized in the freeze-dryer under the abovementioned operating conditions, and the others were kept at −18 °C until extraction. The lyophilization yield for fish meat was 30.8%.

Next, the lyophilizates of the chicken hearts and fish meat were placed in amber glass jars, hermetically sealed using the lids, and kept in a refrigerator (at 4 °C) until use when they were milled to fine powders using an electric grinder (Titan Mil 300 DuoClean; Grupo Cecotec Innovaciones S.L., Valencia, Spain).

The oil press cakes of rapeseed, sunflower, pumpkin, and walnut were received by a donation from Taf Presoil S.R.L. (Luncani, Romania) as pellets, 1 kg of each type, and kept in a refrigerator (at 4 °C) up to extraction when they were milled with an electric grinder (Titan Mil 300 DuoClean; Grupo Cecotec Innovaciones S.L., Valencia, Spain) to fine powders.

### 2.2. Reagents and Standards

The HPLC-grade methanol (34860), HPLC-grade 2-propanol (34863), HPLC-grade ethanol absolute (34852-M), and CoQ10 standard (C9538) were acquired from Sigma-Aldrich Co. (St. Louis, MO, USA) while the 2-propanol pure p.a. (117515002) from was acquired Chempur (Piekary Śląskie, Poland).

### 2.3. Preparation of 2-Propanol Extracts

**Figure 1:**
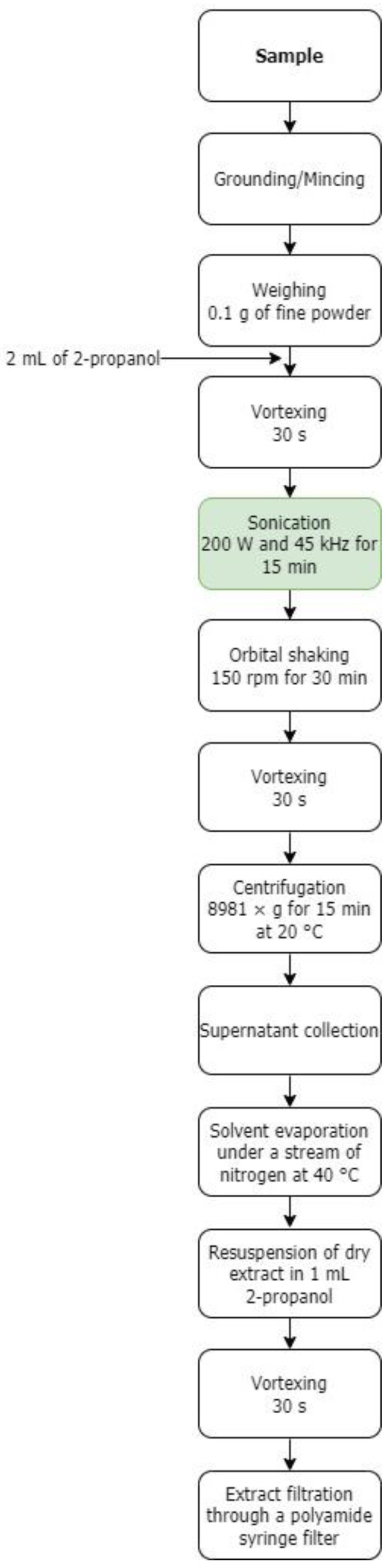
Workflow of the proposed extraction procedure: ultrasonic extraction with 2-propanol.

This was carried out using the slightly modified method of Stiff et al. [23], which consisted of direct extraction with 2-propanol, introducing an additional sonication step as recommended by Zu et al. [25] (see Figure 1). First, into a 15 mL conical centrifuge tube, 0.1 g of minced/finely ground sample was weighted to the nearest 0.1 mg (analytical balance ABJ-220-4NM; Kern & Sohn GmbH, Balingen, Germany). Next, 2 mL of 2-propanol was added and vortexed for 30 s (vortex mixer 6776; Corning Life Sciences, Monterrey, Mexico).

The mixture was sonicated (ultrasonic bath USC 300 THD; VWR International, Singapore, Malaysia) at 200 W intensity and 45 kHz frequency for 15 min, then orbitally shaken (orbital shaker 3005; GFL Gesellschaft für Labortechnik mbH, Burgwedel, Germany) at 150 rpm for 30 min, vortexed for 30 s, and centrifuged (benchtop centrifuge Universal 320 R; Andreas Hettich GmbH & Co. KG, Tuttlingen, Germany) at 8981× *g* (9000 rpm) for 15 min at 20 °C, and the supernatant was collected in a weighted test tube.

The solvent was evaporated under a stream of nitrogen at 40 °C (thermoblock TA 120 P2; FALC Instruments S.R.L., Treviglio, Italy) until a constant weight was obtained (mass change not exceeding 1 mg).

The dried extract was resuspended in 1 mL 2-propanol, vortexed for 30 s, filtered through a polyamide syringe filter (0.45 µm pore size, 25 mm diameter), and kept at −18 °C until chromatographic analysis.

The extraction was performed in triplicate for each sample.

### 2.4. HPLC-DAD Analysis of the Extracts

Separation of CoQ10 was performed on a Kinetex XB-C18 column (150 mm L × 4.6 mm ID × 5 μm particle size; Phenomenex, Torrance, CA, USA) using a mixture of methanol/2-propanol/ethanol (70:15:15, *v*/*v*/*v*) as mobile phase in isocratic elution and a liquid chromatography system (1200 HPLC; Agilent Technologies Inc., Palo Alto, CA, USA) equipped with a diode array detector (DAD) according to a previous method described by Mattila and Kumpulainen [14], Souchet and Laplante [15], Ercan and El [29], Tobin et al. [30], and Román-Pizarro et al. [18], with minor modifications. The system also included a quaternary pump, a degasser, an autosampler, and a thermostatted column compartment.

Twenty microliters of extract were injected into the HPLC system to perform the instrumental analysis. The mobile phase flow rate was programmed to 1.2 mL/min and the column oven temperature was programmed to 25 °C. The DAD was set to 275 nm, and data acquisition was performed for 15 min using ChemStation software (Rev B.04.02 SP1; Agilent Technologies Inc., Palo Alto, CA, USA).

For calibration curve construction, a stock solution of CoQ10 was prepared at 1000 µg/mL in 2-propanol and subsequently diluted with the same solvent to prepare six working solutions at 0,25, 1.0, 50, 100, 150, and 200 µg/mL. The stock solution and working standards were stored at −18 °C in the dark for further use.

Before injection into HPLC, each sample extract or working standard was filtered through polyamide syringe filters (0.45 µm pore size and 25 mm diameter). CoQ10 was identified by comparing its retention time to the standard’s one (studied under the same conditions). The results were expressed in µg CoQ10/g sample. All samples and working standards were analyzed in triplicate.

**Figure 2:**
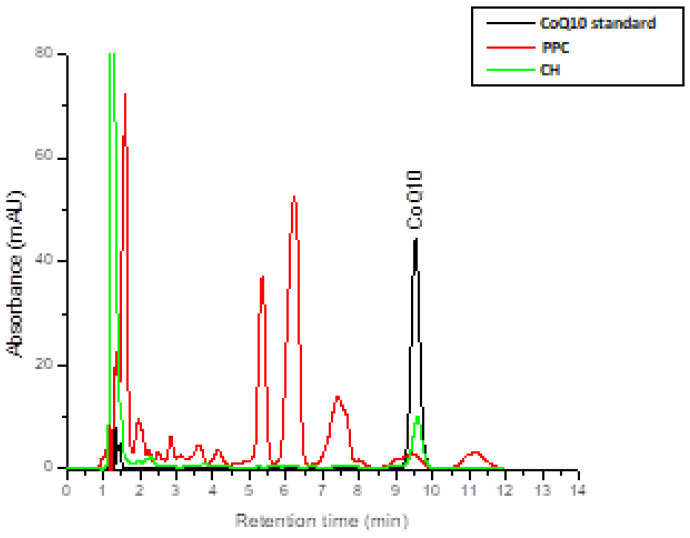
Overlay of chromatograms obtained by chromatographic separation of pumpkin press cake (PPC) and chicken heart (CH) extracts with the CoQ10 standard (detection wavelength: 275 nm).

Figure 2 shows the overlap of some chromatograms, such as that of the RPC (with the highest content of CoQ10 from all vegetable matrices) and CH (the animal matrix where CoQ10 was detected), with that of the CoQ10 standard.

### 2.5. Statistical Analysis

The statistically significant difference between the mean CoQ10 levels of oil press cakes, fish meat, and chicken hearts was determined by performing one-way analysis of variance (ANOVA) with a post hoc Tukey’s test at a 95% confidence level (*p* < 0.05) using Minitab statistical software (version 19.1.1; LEAD Technologies, Inc., Charlotte, NC, USA).

## 3. Results and Discussion

HPLC with DA detection at 275 nm is the most common technique to estimate CoQ10 in foods, generally using calibration with an external standard [9]. For example, in 2000, it was used by Mattila et al. [13] to measure CoQ10 levels in pork heart, beef meat, and Baltic herring flesh by applying direct extraction with *n*-hexane/ethanol (5:1, *v*/*v*). One year later, the same authors [14] employed direct ethanol/*n*-hexane (2:5, *v*/*v*) extraction for samples such as cauliflower, potato, tomato, carrot, pea, bean, orange, clementine, apple, blackcurrant, lingonberry, strawberry, reindeer meat, pork heart, beef meat, beef heart, chicken meat, pollack flesh, hen’s egg, skimmed milk (1.5% fat), and yogurt while for samples such as rapeseed oil, pork liver, beef liver, Emmental cheese, and Edam cheese, saponification with an aqueous potassium hydroxide solution took place prior to *n*-hexane extraction. The preparation protocol proposed by Souchet and Laplante in 2007 [15] to determine the level of CoQ10 in mackerel flesh, herring flesh, mackerel heart, and herring heart included sample homogenization with sodium dodecyl sulfate and a sodium chloride solution followed by extraction with ethanol/hexane (2:5, *v*/*v*). After two years, Laplante et al. [16] applied the same method to quantify CoQ10 in whole mackerel and whole herring; the oils of mackerel and herring, which were also assessed, were extracted by enzymatic hydrolysis from lyophilized fish samples using Protamex^TM^ and supercritical CO_2_ and were then dissolved in 2-propanol before HPLC analysis. In 2012, Xue et al. [17] developed a method for dosing the level of CoQ10 in rape, apricot, tea, and mixed bee pollen by applying an online clean-up of accelerated extraction using absolute ethanol. The method of Mattila and Kumpulainen [14] was also used by Román-Pizarro et al. [18] in 2017 for samples such as parsley, spinach, avocado, peanut, pistachio, pork liver, and beef liver, utilizing a mixture of ethanol/*n*-hexane (2:5, *v*/*v*) for extraction. In 2018, Mandrioli et al. [19] investigated the level of CoQ10 in whole cow milk using the internal standard method (CoQ9); the sample was first saponified with an ethanolic potassium hydroxide solution and a pyrogallol solution, then extracted with petroleum ether/diethyl ether (9:1, *v*/*v*). Although efficient, these extraction protocols either use toxic solvents, too many or in too large quantities or require too many steps for extraction, thus making them challenging to implement in the large-scale extraction of CoQ10. Therefore, this study proposes a simple extraction procedure that uses a single solvent, 2-propanol, which is non-toxic and environmentally friendly; the recovered solvent can be used for repeated extractions within the same matrix, thus reducing the production costs.

The results of linearity and spike-and-recovery experiments carried out in this study are shown and discussed in Section 3.1 and Section 3.3 of this paper, respectively; those regarding the determination of CoQ10 content in rapeseed press cake (RPC), sunflower press cake (SPC), pumpkin press cake (PPC), linseed press cake (LPC), walnut press cake (WPC), hempseed press cake (HPC), whole fish (WF), lyophilized whole fish (LWF), chicken hearts (CH), and lyophilized chicken hearts (LCH) are in Section 3.2.

### 3.1. Linearity and Measuring Range, Limits of Detection (LOD), and Quantification (LOQ)

Linearity and Measuring Range. The linearity of an analytical method is the interval in the measurement range in which the output signal of the analyte linearly correlates with its determined concentration. A measuring range is a set of values (analyte concentrations) where a measuring instrument’s error is less than the value assumed [31]. The linearity check should confirm that the analytical method produces a linear response; as an acceptance criterion of testing, the regression coefficient must be more than 0.99 over the measuring range [20].

Six standard solutions of CoQ10, with concentrations of 0.25, 1.0, 50, 100, 150, and 200 µg/mL, were analyzed using the HPLC-DAD method described in Section 2.4, with three successive injections performed for each. The calibration curve was constructed by plotting the average value of each standard’s peak area against its concentration (µg/mL). However, the regression equation (y=16.759x−40.466) was linear only in the 1–200 µg/mL range, with it having a good coefficient of regression (R^2^ = 0.9974). An almost similar linear range (0.25–200 µg/mL) was reported by Xue et al. [17] when the HPLC-DAD method mentioned above was used to quantify CoQ10 in bee pollen.

Limit of Detection (LOD) and Limit of Quantification (LOQ). The lowest concentration (smallest amount) of an analyte that can be detected with statistically significant certainty is known as the LOD. The amount, or the smallest concentration, of an analyte that can be determined using a specific analytical procedure with an assumed accuracy, precision, and uncertainty is the LOQ [31].

The measurement results obtained for standard solutions of CoQ10 with the three lowest concentrations (0.25, 1.0, and 50 µg/mL) were used to determine the detection and quantification limits, as described by Konieczka and Namiesnik [31] and Michiu et al [32]. For this purpose, another calibration curve was outlined just based on these data; then, the LOD and LOQ were calculated with the formulas below (1), (2):

(1) $$LOD\left(\mu g/mL\right)=3.3\times \frac{\sigma_{a}}{b}$$

(2) $$LOQ\left(\mu g/mL\right)=10\times \frac{\sigma_{a}}{b}$$

where σ*_a_* is the intercept’s standard deviation and *b* is the slope.

Values of 0.22 µg/mL for LOD and 0.65 µg/mL for LOQ were thus obtained, comparable with those reported by Mandrioli et al. [8] (0.35 µg/mL for LOD and 1.18 µg/mL for LOQ) with the HPLC-DAD method that they used. The following conditions must be met to verify the LOD estimated: $LOD<C_{min}$ (concentration of the lowest standard used for the LOD determination) and $10\times LOD>C_{min}$ [31]. These were achieved in the present study.

### 3.2. Levels of CoQ10 in Food By-Products and Waste

The CoQ10 content was determined for all three sample replicates on two consecutive days (see Table 1). The intra- and inter-day repeatability of measurements, in terms of relative standard deviation (RSD), was calculated for each sample by the following Formula (3):

(3) $$RSD\left(\%\right)=\frac{\sigma \times 100}{X_{mean}}$$

where σ is the standard deviation calculated using the STDEV function in Excel, and $X_{mean}$ is the mean CoQ10 level of the three replicates.

To estimate the results precisely, the mean value of all six measurements (three on the 1st day of analysis and three on the 2nd day) and the pooled standard deviation (*σ_pooled_*) was calculated (4) for each sample.

(4) $$\sigma_{pooled}=\sqrt{\frac{\left(2\times {\sigma_{1}}^{2}\right)+\left(2\times {\sigma_{2}}^{2}\right)}{3+3}}$$

where $\sigma_{1}$ is the standard deviation of the three values obtained on the 1st day of measurement, 2 means the degrees of freedom (3-1), and 3 is the number of measurements performed daily.

Next, the pooled relative standard deviation (*RSD_pooled_*) was computed for each sample with the below Formula (5):

(5) $${RSD}_{pooled}\left(\%\right)=\frac{\sigma_{pooled}\times 100}{X_{mean}}$$

where $\sigma_{pooled}$ is the pooled standard deviation calculated above, and $X_{mean}$ is the mean CoQ10 level of all six replicates (from both measurement days).

Oil press cakes. The richest source of CoQ10 is PPC (84.80 µg CoQ10/g sample), followed by RPC (56.77 µg CoQ10/g sample). On the other hand, there is no significant difference in the CoQ10 level of RPC and LPC (53.70 µg CoQ10/g sample) or between that of LPC and SPC (49.29 µg CoQ10/g sample). The lowest concentration of CoQ10 was found in WPC (36.56 µg CoQ10/g sample), while in HPC, it was not detected. Most studies have only focused on determining CoQ10 in raw materials and oils [9,34], and, to our knowledge, this is the first one conducted on oil press cakes. In the study of Rodríguez-Acuña et al. [34], CoQ10 was isolated by solid-phase extraction (SPE) from crude oil of rapeseed and refined oil of sunflower, showing levels of 46.4 and 8.7 µg/g, respectively.

Fish meat. CoQ10 was not detected in the WF and LWF samples analyzed in this study. In contrast to our findings, Souchet and Laplante [15] found concentrations of 18.6 µg CoQ10/g raw meat and 88.4 µg CoQ10/g lyophilized meat in whole mackerel, and 9.9 µg CoQ10/g raw meat and 50.9 µg CoQ10/g lyophilized meat in whole herring. Given that mackerel and herring are fatty fishes (above 8% fat content) and the rainbow trout investigated here is a mid-fat fish (2–8% fat content) [35], it can be concluded that the CoQ10 content in fish is proportional to its fat content.

In the study of Laplante et al. [16], the performance and production costs of supercritical CO_2_ extraction versus enzymatic hydrolysis followed by centrifugation were compared to recover the oils and CoQ10 from mackerel and herring. The enzymatic hydrolysis process gave higher oil and CoQ10 yields with both fish species and generated lower production costs. A 65 µg/g concentration was found in mackerel oil and 275 µg/g was found in herring oil.

Chicken hearts. The highest content of CoQ10 was found in the chicken hearts, with a level of 114.39 µg CoQ10/g for the CH sample and 383.25 µg CoQ10/g for the LCH sample. Through lyophilization, the CoQ10 amount recovered from the chicken hearts was approximately 3.3 times higher. Although expensive, freeze-drying reduces the weight and volume of waste, providing better storage stability and higher extraction yield for CoQ10. The result obtained for CH is consistent with those of Kubo et al. [22], who reported a similar concentration, 107 µg CoQ10/g, in raw chicken hearts. However, CoQ10 is quickly oxidized when exposed to light or in contact with air [36], with its stability being affected by storage time and conditions. Therefore, extracting CoQ10 as close as possible to the moment the food matrix has become waste is essential.

In a more recent study, Villanueva-Bermejo and Temelli (2011) [37] investigated the level of CoQ10 in oil recovered from chicken hearts by supercritical CO2 extraction. They found a concentration of 670 µg/g in the oil obtained during the constant extraction rate and 2310 µg/g in the one obtained during the falling extraction rate. Hence, more CoQ10 can be recovered from a food matrix by extracting its oil. However, supercritical fluid technology is expensive due to the high investment costs [38]. Oil extraction using a green solvent followed by solvent evaporation could be, thus, a less expensive alternative.

### 3.3. Accuracy (Trueness and Precision)

The ISO 5725-4:2020 standard [39] uses two terms, “trueness” (the closeness of agreement between the expected value of a measurement result and the actual value) and “precision” (the closeness of agreement between independent measurement results obtained using predetermined conditions), to describe the accuracy of a measurement method.

The analytical method trueness, reported as percentage recovery (see Table 2), was assessed on samples of PPC and CH spiked with the same volume of CoQ10 standard solution at three different concentrations so that in final extracts, it reached 0.5, 1.5, and 5.0 µg CoQ10/mL, which corresponded to 5, 10, and 50 µg CoQ10/g sample, respectively. The recovery rate was calculated using the following Formula (6) [40]:

(6) $$Recovery\;rate\left(\%\right)=\frac{C_{spiked}}{C_{expected}}\times 100$$

where *C_spiked_* is the concentration of CoQ10 found in the spiked sample (µg CoQ10/mL) and *C_expected_* is the expected (theoretical or calculated) CoQ10 concentration in the spiked sample (µg CoQ10/mL).

(7) $$C_{expected}(\mu g\;CoQ10/mL)=C_{unspiked}+C_{added}$$

where *C_unspiked_* is the CoQ10 concentration found in the unspiked sample (µg CoQ10/mL) and *C_added_* is the concentration of CoQ10 added to the spiked sample (µg CoQ10/mL).

For all analytes within the scope of a method, mean recoveries from initial validation should fall between 70 and 120%, with the associated repeatability (RSD) of less than or equal to 20% [41]. The recovery of an analyte depends on the matrix complexity, the sample preparation procedure, and the analyte concentration [42]. A too-low recovery underestimates the calculated LOD [31].

The average recovery rate of CoQ10 from the PPC matrix ranged between 100.9% and 116.0% (see Table 2), while from the CH sample, it ranged from 99.3 to 106.9%, within the European Commission SANTE 11312/2021 guideline requirements (mean 70–120%; RSD ≤ 20%) [41]. Similar recovery rates (100.4–113.0% for mackerel red flesh; 98.0–100.4% for herring’s heart) were reported by Souchet and Laplante [15] in fish samples spiked with 1–15 µg CoQ10 by using an HPLC-DAD method. The percentage RSD between the recovery values (see Table 2), which indicates the precision of our method, was in the range of 0.05 and 0.2% for the PPC sample and, for the CH one, it was between 0.5 and 0.7%. All these results meet the acceptance criteria mentioned in the previous paragraph.

## 4. Conclusions

A valuable analytical method has been developed here to determine CoQ10 in oil press cakes, fish meat, and chicken hearts by modifying a previous extraction procedure-introducing an additional sonication step. Briefly, this consisted of sample homogenization with 2-propanol, followed by sonication, short maceration, centrifugation, supernatant collection, solvent evaporation, dry-extract resuspension in 2-propanol, and filtration before HPLC analysis. As a result, a very good recovery rate for CoQ10, with excellent repeatability, was achieved. In addition, the solvent used, 2-propanol, is environmentally friendly, economically viable, and feasible for scaling up the production of CoQ10. The highest level of CoQ10 was found in CH and then in PPC. Therefore, these matrices can be used as raw materials to extract CoQ10 for further applications in different industries (foods, supplements, pharmaceutics, and cosmetics).

This study’s findings provide information regarding CoQ10-rich food by-products and waste, a green extraction procedure, and an HPLC method for CoQ10 analysis, which might be helpful to natural extract producers. Future work will focus on lipid fraction extraction from these matrices using 2-propanol to characterize them towards obtaining natural dietary supplements based on CoQ10.

## 5. Patents

Patent application A/00138 from 24 March 2023: “Process for the preparation of some natural dietary supplements based on coenzyme Q10”. Inventors: Cristina-Anamaria Semeniuc, Andersina-Simona Podar, Sonia-Ancuța Socaci, Floricuța Ranga, Simona-Raluca Ionescu, Maria-Ioana Socaciu, Melinda Fogarasi, Dan-Cristian Vodnar, and Anca-Corina Fărcaș

## Figures and Tables

**Table 1:** CoQ10 levels in the oil press cakes, fish meat, and chicken hearts.

Sample/MC (%)	1st Day of Measurement		2nd Day of Measurement		Mean ± σ_pooled_ (µg CoQ10/g Sample)	RSD_pooled_ (%)
	Mean ± σ (µg CoQ10/g Sample)	RSD (%)	Mean ± σ (µg CoQ10/g Sample)	RSD (%)		
RPC/10.4%	57.06 ± 2.316	4.1	56.47 ± 1.717	3.0	56.77 ± 1.664 ^d^	2.9
SPC/9.8%	48.80 ± 1.386	2.8	49.78 ± 1.480	3.0	49.29 ± 1.171 ^e^	2.4
PPC/9.9%	84.30 ± 0.978	1.2	85.29 ± 2.151	2.5	84.80 ± 1.364 ^c^	1.6
LPC/10.7%	53.50 ± 0.913	1.7	53.89 ± 0.906	1.7	53.70 ± 0.743 ^de^	1.4
WPC/9.8%	36.56 ± 0.845	2.3	36.56 ± 0.352	1.0	36.56 ± 0.528 ^f^	1.4
HPC/11.2%	n.d.	-	n.d.	-	n.d.	-
WF/69.0%	n.d.	-	n.d.	-	n.d.	-
LWF/3.4%	n.d.	-	n.d.	-	n.d.	-
CH/77.0%	119.95 ± 11.141	10.0	116.83 ± 11.419	9.8	114.39 ± 9.211 ^b^	8.1
LCH/3.2%	384.52 ± 0.680	0.2	381.97 ± 0.872	0.2	383.25 ± 0.639 ^a^	0.2

MC—moisture content (%) determined using oven drying at 103 °C to a constant weight [33]; RPC—rapeseed press cake; SPC—sunflower press cake; PPC—pumpkin press cake; LPC—linseed press cake; WPC—walnut press cake; HPC—hempseed press cake; WF—whole fish; LWF—lyophilized whole fish; CH—chicken hearts; LCH—lyophilized chicken hearts; n.d.—not detected; σ—standard deviation; RSD—relative standard deviation; σ_pooled_—pooled standard deviation; RSD_pooled_—pooled relative standard deviation. Results are expressed as mean ± standard deviation of triplicate data (*n* = 3). Different letters in the row indicate a statistically significant difference at *p* < 0.05 (Tukey’s test).

**Table 2:** Recovery rates (trueness values) of CoQ10 from samples of PPC and CH with RSDs (precision values).

Sample	Spiking Concentration (µg CoQ10/mL)	Found Concentration (µg CoQ10/mL)	RSD (%)	Recovery Rate (%)	RSD (%)
PPC	-	12.05 ± 0.042	0.4	-	-
	0.5	14.56 ± 0.042	0.3	116.0 ± 0.054	0.05
	1.5	15.63 ± 0.042	0.3	115.4 ± 0.053	0.05
	5.0	17.21 ± 0.084	0.5	100.9 ± 0.242	0.2
CH	-	14.29 ± 0.0	0.0	-	-
	0.5	15.66 ± 0.084	0.5	105.9 ± 0.569	0.5
	1.5	16.88 ± 0.127	0.7	106.9 ± 0.802	0.7
	5.0	19.15 ± 0.127	0.7	99.3 ± 0.656	0.7

PPC—pumpkin press cake; CH—chicken hearts; RSD—relative standard deviation. The results are expressed as the mean ± standard deviation of the triplicate data (*n* = 3).

## Data Availability

The data presented in this study are available on request from the corresponding author.
